# Antimicrobial Resistance: The Next Probable Pandemic

**DOI:** 10.31729/jnma.7174

**Published:** 2022-02-28

**Authors:** Ashima Gautam

**Affiliations:** 1 Nepal Medical College and Teaching Hospital, Attarkhel, Jorpati, Kathmandu, Nepal

**Keywords:** *antibiotics*, *drug resistance*, *global health*, *medical students*, *pandemic*

## Abstract

As the world still mourns the victims of the pandemic caused by Severe Acute Respiratory Syndrome Coronavirus-2, another pandemic that is expected to kill millions of people in less than a century, is already brewing. In the distant future, the global, mostly silent pandemic of antimicrobial resistance is increasingly claiming the lives of patients on hospital floors. Unfortunately, the global health community is now gradually and progressively facing the silently emerging pandemic that could endanger some of the most significant advances in modern medicine. Medical students as future physicians, have the potential to help address this problem sustainably keeping in mind that today's medical professionals will hand over the baton to them and hope for a greater improvement in antimicrobial resistance and antibiotic usage. Thus, the next generation of doctors must be better prepared to use antimicrobials more sparingly and appropriately.

## HISTORY

To quote the former director of the Centers for Disease Control and Prevention (CDC), Tom Frieden, "If we use antibiotics when not needed, we may not have them when they are most needed."

The introduction of antibiotics into clinical use was arguably the greatest medical breakthrough of the 20th century. In addition to treating infectious diseases, antibiotics made many modern medical procedures possible, including cancer treatment, organ transplants, and open-heart surgery. The development of anti-infective drugs and the underlying concept of chemotherapy is widely accredited to Paul Ehrlich, who deployed the synthetic arsenic-based pro drugs salvarsan (salvation arsenic) and neo-salvarsan circa 1910 to treat Treponema pallidum. Alexander Fleming's accidental discovery of penicillin in 1928 started the golden age of natural product antibiotic discovery that peaked in the mid-1950s.^1^ In just over 100 years, antibiotics have drastically changed modern medicine and extended the average human lifespan by 23 years.^2^

## ANTIMICROBIAL RESISTANCE

"The time may come when penicillin can be bought by anyone in the shops. Then there is the danger that the ignorant man may easily underdose himself and by exposing his microbes to non-lethal quantities of the drug, make them resistant", said Alexander Fleming, speaking in his Nobel Prize acceptance speech in 1945. As predicted almost 70 years ago by the man who discovered the first antibiotic, drug resistance is upon us.^3^

The success of antimicrobials has been impressive. At the same time, however, excitement about them has been tempered by a phenomenon called antimicrobial resistance (AMR). This is a problem that surfaced not long after the introduction of penicillin and now threatens the usefulness of these significant medicines.^2^

Antimicrobial Resistance (AMR) occurs when bacteria, viruses, fungi, and parasites change over time and no longer respond to medicines making infections harder to treat and increasing the risk of disease spread, severe illness, and death. AMR occurs naturally over time, usually through genetic changes because of evolution by natural selection. As a result of drug resistance, antibiotics and other antimicrobial medicines become ineffective and infections become increasingly difficult or impossible to treat.^4^

In 2019, a United Nations (UN) report estimated that drug-resistant microbes could lead to ten million deaths per year, and cost the world $100trn, by 2050.^5^

## STATUS IN NEPAL

A 2014 WHO report includes data from Nepal on antibiotic resistance rates for six combinations of bacterial pathogens and antibiotics. The bacteria were E. coli, S. aureus, non-typhoidal Salmonella, Shigella spp., K. pneumonia, and N. gonorrhea. Out of the 140 isolates included, 64 percent of E. coli isolates were resistant to fluoroquinolones and 38 percent were resistant to third-generation cephalosporins. Smaller data sets showed methicillin-resistant Staphylococcus aureus (MRSA) ranging from 2 to 69 percent. K. pneumonia showed resistance to third-generation cephalosporins of 0 to 48 percent, while no resistance to carbapenems was detected.^6^

Several studies of antibiotic prescribing patterns in Nepal showed most patients were unnecessarily prescribed more than one antibiotic concurrently without bacterial confirmation or susceptibility testing.^7^ Antibiotics used against bacteria in urine, pus, and blood of infected patients in several Nepali hospitals were effective in curing only 50% of cases whereas the other 50% showed no response.^8^

It is known that antibiotics are easily available over the counter in pharmacies without a prescription. Therefore, self-medication is very common in Nepal and most people do not comply with the treatment prescribed by the physician.^6^ Most patients fail to follow a full course of treatment and usually stop taking medicines after 2-3 days when symptoms start to subside. They then store the leftover medicines for future self-medication, thereby prolonging the duration of illness and speeding up the rate of development of resistance. Furthermore, some practitioners are found to be prescribing broad-spectrum antibiotics even when the problem can be overcome by a narrow spectrum drug. Antibiotics are prescribed for colds, coughs, and diarrhea that are likely to heal following a simple course of supportive treatment.^7^

## ANTIBIOTICS STEWARDSHIP PROGRAM (ASP)

One of many reasons why antibiotic use is so high is that there is a poor understanding of the differences between bacteria, viruses, and other pathogens, and the proper use and value of antibiotics. Antibiotics are very often prescribed for no useful purpose. Too many antibiotics are prescribed for viral infections such as colds, flu, and diarrhea. ^3^

Antibiotics stewardship program (ASPs) is therefore a systemic effort to educate and persuade prescribers of antimicrobials to follow evidence-based prescribing to stop antibiotics overuse and antimicrobial resistance. As Antibiotics are among the most commonly prescribed drugs in hospitals, staying up to date on the latest clinical guidelines and local antimicrobials resistance pattern is always necessary.

## MEDICAL STUDENTS AND ANTIMICROBIAL RESISTANCE

Studies have detected that prescribers display significant gaps in knowledge and inappropriate practices regarding antibiotics and resistance, but it is not known whether these are generated during professional practice or derived from the undergraduate stage of their education.^9^

There were various surveys conducted in different parts of the world among medical students using questionnaires as research tools to assess knowledge on emerging resistance of superbugs. Although medical students recognize the issue of resistance, they lack basic knowledge regarding AMR. The studies showed that the majority of the medical students had sufficient knowledge of the mechanism of action of antibiotics but the vast majority of the respondents demanded more education on antibiotics use and its prescription.^10^ Furthermore, only a third of the students felt confident in prescribing antibiotics and they tend to feel more confident in the diagnosis of infections rather than choosing an accurate therapeutic plan. The results may not be representative of all medical students however; all this suggests the need to improve education and awareness on antibiotics, resistance and treatment of infections from the dawn of medical journey.

## ROLE OF MEDICAL STUDENTS IN LIMITING THE SPREAD OF RESISTANCE

Taking into consideration the importance of primary education on one's career, medical students need to be aware about global health issues such as AMR from the pre clinical years itself. For that reason, faculties in medical schools along with the professionals should be able to identify and fix gaps in their curriculum as per the forthcoming needs. Moreover, an antimicrobial resistance program (ASP) should be attended by undergraduates in various institutions to grasp knowledge about resistance trends and drug sensitivity for specific organisms. Such a program can set about accurate antimicrobial choices. Once the students have attained a required understanding of the field, they can help educate fellow students in medical schools. In addition to promoting change in their schools and hospitals, medical students can further play a key role as educators and advocate in their local communities as well. They can also devote some time to form a forum dedicated to educating peers and create an organization of doctors, scientists and professionals to understand core issues in this field. One such organization exists in Nepal as Alliance against Antimicrobial resistance (AAAMR). Since its establishment, AAAMR has been working with some medical and health care students conducting awareness campaigns among medical professionals as well as among the general population. Involvement of medical students in such organizations will engage them in research and subsequently generate adequate knowledge leading to wise prescription of antimicrobial drugs further in their medical career.

Hence, by influencing the next generation of physicians before they have developed the bad habits of prescribing antibiotics for the wrong reasons, overprescribing can be drastically decreased.

## WORLD ANTIMICROBIAL AWARENESS WEEK

The World Antibiotic Awareness Week (WAAW), also known as World Antimicrobial Awareness Week, is an annual event conducted by the World Health Organization (WHO). Every year in November, initiatives are taken in many countries around the world to increase awareness of global antimicrobial resistance and to encourage best practices among the general public, health workers, and policymakers to avoid the further emergence and spread of drug-resistant infections. The first-ever public campaign was done in Europe but as the campaigns continued to grow and spread, more countries kept joining the initiative.^11^ Even this year, the world antimicrobial awareness week has been celebrated all over the world with the theme 'Spread Awareness, Stop Resistance'.^12^

## WAYS FORWARD

It is a boon that effective antimicrobials have been one of the pillars allowing us to live longer, be healthier, and benefit from modern medicine. We now take it for granted that many infections are treatable with antibiotics. Surgery, cancer treatment, intensive care, transplant surgery, even simple wound management would all become much riskier, more difficult options if antibiotics could not be used to prevent infection, or treat infections. As a medical student, one could comprehend the fact that the use and misuse of antimicrobials might result in rapidly increasing levels of its resistant bugs leading to a loss of drugs efficacy. Therefore, it's high time we realize the magnitude of the problem and collectively join the battle against superbugs. Unless we take significant actions to improve efforts to prevent infections and also change how we produce, prescribe and use antibiotics, the world will lose more and more of the global public health goods and the consequences will be devastating.
